# Health utility of patients with advanced gastrointestinal stromal tumors (GIST) after failure of imatinib and sunitinib: findings from GRID, a randomized, double-blind, placebo-controlled phase III study of regorafenib versus placebo

**DOI:** 10.1007/s10120-014-0391-x

**Published:** 2014-06-24

**Authors:** Chris D. Poole, Mark P. Connolly, Jane Chang, Craig J. Currie

**Affiliations:** 1 Institute of Primary Care and Public Health, Cardiff University, Wales, UK; 2 Global Market Access Solutions, St.-Prex, Switzerland; 3 Pharmaco Economics and Pharmaco Epidemiology Unit, Department of Pharmacy, University of Groningen, Antonius Deusinglaan 1, 9713 AV Groningen, Netherlands; 4 Bayer HealthCare, 100 Bayer Blvd, Whippany, NJ 07981 USA

**Keywords:** Quality of life, Gastrointestinal stromal tumour, EuroQol-5D, Health state utilities

## Abstract

**Background:**

In this analysis we report patients with advanced gastrointestinal stromal tumors (GIST) refractory to imatinib and sunitinib therapy as derived from the EuroQol-5D (EQ-5D) for progression-free (PF) and progressive disease health status.

**Methods:**

Data were analyzed from a phase III trial conducted at 57 hospitals in 17 countries (trial registration number, NCT01271712). Patients with advanced GIST were randomized (2:1) to receive blinded treatment using oral regorafenib 160 mg daily or placebo, plus best supportive care (BSC) in both groups, for the first 3 weeks of each 4-week cycle. EQ-5D-3L was administered on day 1 of each cycle before contact with their physician and before any study-related procedures. The effect of disease progression on the utility of EQ-5D was tested with paired-samples comparison and general linear mixed modeling (GLMM).

**Results:**

One hundred and eighty five patients [93 % of the intention-to-treat (ITT) population] completed 803 EQ-5D questionnaires: 77.7 % in progression-free (PF) state, 6.5 % at progression, 13.9 % following first progression, and 1.9 % after second progression. Mean baseline utility was 0.767 (SD 0.221) with no significant between-group differences for active treatment and BSC. The first post-progression health state was 0.647 (SD 0.343), suggesting significantly impaired health-related quality of life after confirmed disease progression showed a decrease of −0.120 (paired samples *t* test, *p* = 0.001). GLMM showed no effect of study treatment or cycle number on utility.

**Conclusions:**

We demonstrate a significant and clinically meaningful difference in health state utility values between PF and progression. Utility values remained stable over successive regorafenib cycles after controlling for disease status and treatment type.

## Background

Gastrointestinal stromal tumors are probably the most common type of soft tissue sarcoma, likely arising from precursor cells of the interstitial cells of Cajal. They may arise anywhere along the gastrointestinal tract, with approximately 60–70 % occurring in the stomach and 20–30 % in the small intestine [1, 2]. The majority of cases are diagnosed in individuals more than 60 years of age , although GIST has been reported in all age groups [3–5]. Molecular diagnosis of GIST can confirm suspected diagnosis, with the majority of subjects expressing KIT receptor tyrosine kinase (CD117) [6]. The incidence of cases of GIST has been reported to vary from 6.5 to 14.5 per million, with slightly higher rates observed in males [3–5]. At diagnosis, approximately 10–20 % of the tumors are metastatic [7, 8]. Symptomatic GIST normally presents as bleeding, fatigue, or abdominal discomfort [2, 4].

Nonmetastatic patients are treated with surgery. Adjuvant or neoadjuvant imatinib is administered when the risk of GIST recurrence is considered significant [9]. Approximately 60 % of the patients with nonmetastatic GIST are cured by surgery [10]. For GIST patients with metastatic or unresectable disease, molecular targeted therapies involving tyrosine kinase inhibitors are recommended. Even in the absence of clinical benefit, these agents are recommended as part of best supportive care (BSC). The national comprehensive cancer network guidelines recommended that the decision to continue tyrosine kinase therapy is dependent on tolerability, benefit–risk assessment, and quality of life [4]. In metastatic disease, limited response to cytotoxic chemotherapy has been observed and thus it is not recommended in patients with GIST [4].

Increasingly the patient experience is viewed as an important outcome for cancer patients where societal preferences for quantity and quality of life are recognized [11]. In GIST patients with advanced disease, maintaining quality of life is particularly important as many patients survive for many years with the targeted treatments, and current therapies used to treat GIST can cause side effects known to impair quality of life (QoL) [12]. This approach illustrates the advantages of measuring the consequences of therapeutic options to better understand whether side-effect profiles of new interventions pose any threat to QoL for progression-free and progressed patients [13].

Considering the importance of patient preference for different health conditions, regulatory agencies allow for the inclusion of QoL data to support efficacy claims [14]. Patient-reported outcomes are also considered by reimbursement authorities for making decisions that influence funding and access to treatment [15]. The construct of patient QoL not only considers the burden associated with a condition, but also the direct impact of therapeutic interventions on an individual’s disease perception and consequently QoL. Because the outcome of interest for many reimbursement agencies is the quality-adjusted life-year, often derived from instruments such as the EQ-5D, it is often included in clinical trials to support future reimbursement [15, 16]. These measures are favored by reimbursement agencies, particularly those that are preference-based health measures such as the EQ-5D, as this allows for the comparison of different health conditions and facilitates reimbursement decision making [17].

The objective of this study was to characterize the health state utility of patients with advanced gastrointestinal stromal tumors (GIST) refractory to imatinib and sunitinib therapy and treated with regorafenib plus best supportive care (BSC) or placebo plus BSC. Specifically, we estimate the health states of patients remaining progression free and those with clinically diagnosed progression obtained from the GRID study. This type of analysis is conducted to inform economic models based on disease status. Future GRID publications will report quality of life data by treatment exposure.

## Methods

### Trial design

The EQ-5D data were obtained from GRID, a randomized, double-blind, placebo-controlled, multicenter, crossover phase III study originally designed to evaluate the efficacy and safety of regorafenib in subjects with histologically proven metastatic or unresectable gastrointestinal stromal tumor not amenable to surgery, radiation, or a combination of different approaches with curative intent. Recruited subjects must have shown objective disease progression or intolerance to imatinib, as well as disease progression while on sunitinib treatment. Further details of selection criteria have been published previously [18]. The primary endpoint was progression-free survival (PFS) per modified RECIST. Overall survival was one of the secondary endpoints, and health-related quality of life (HRQoL) was assessed as an exploratory endpoint. The study was an international trial conducted at 57 study centers in 17 countries. Study centers were selected on the basis of appropriate clinical training and the expertise of the local principal investigator.

### Interventions

**Fig. 1:**
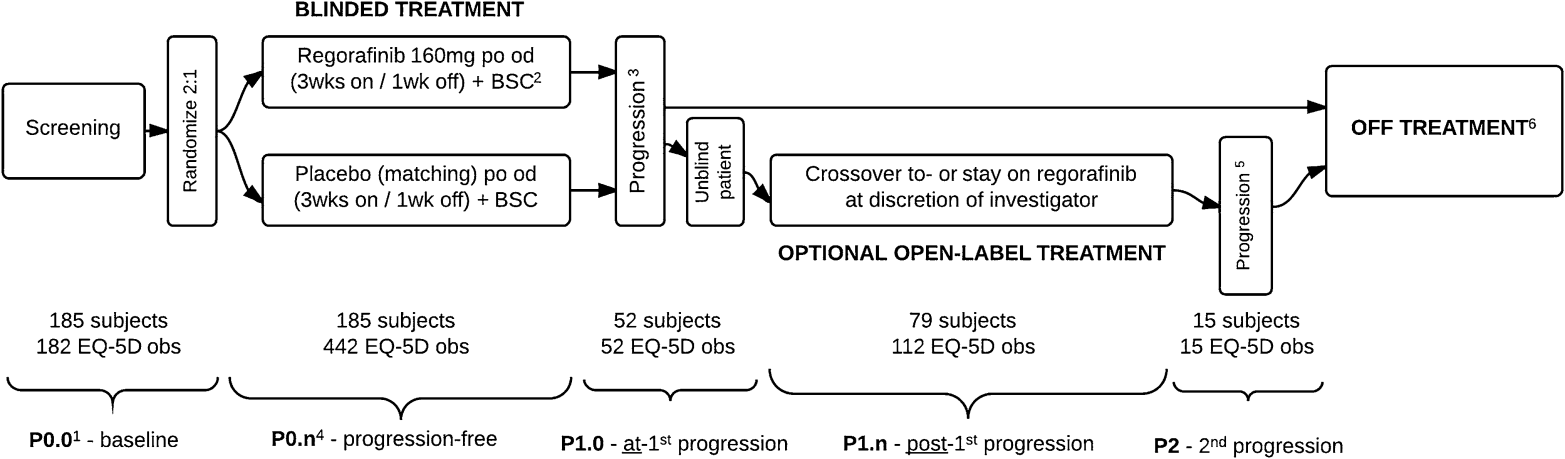
Patient numbers included in the EuroQol-5D (EQ-5D) analysis per study period. Notes: *1* EQ-5D was measured at the beginning of each treatment cycle before the patient consulted a physician and before any study-related procedures were done. *2* Best supportive care includes any method to preserve the comfort and dignity of the patients, and excludes any disease-specific antineoplastic therapy such as any kinase inhibitor, chemotherapy, radiation therapy, or surgical intervention. *3* Confirmed by central radiology review following modified RECIST v1.1 criteria. *4* Where *n* equals cycle number for given progression period. *5* Identified by local investigator/radiologist and not confirmed centrally. *6* Including withdrawals for reasons of toxicity or dropouts for any reason

Screened patients were randomized to receive either oral regorafenib 160 mg daily for 3 weeks of every 4-week cycle or a placebo of identical appearance according to the same regimen in addition to best supportive care (BSC). Patients continued blinded study treatment cycles until disease progression occurred, as confirmed by blinded central radiology review, or withdrawal. Following unblinding, patients receiving placebo who experienced disease progression could be offered open-label regorafenib (crossover option). Those randomized to regorafenib were able to continue open-label regorafenib if the local investigator responsible for their care believed that treatment with regorafenib was providing clinical benefit to that patient. Patients then continued to receive open-label regorafenib plus BSC according to the same 4-week cyclical regimen until further disease progression had occurred. Following this further progression, treatment with regorafenib was withdrawn and patients were then managed with BSC alone (Fig. 1).

For our purposes the intended aim was to measure health state utility values for disease states irrespective of treatment allocation in the GRID trial; hence, the two populations from the GRID study were combined into a single dataset for deriving utilities. Treatment used at any given cycle during either blinded-, open-label-, or off-treatment period was thus characterized as regorafenib + BSC, placebo + BSC, or off-treatment.

### Clinically defined health states

Disease progression was determined by radiologic tumor assessment using either computed tomography (CT) or magnetic resonance imaging (MRI) according to modified study-specific response evaluation criteria in solid tumors (RECIST) 1.1, the details of which have been published previously [19]. For patients receiving blinded treatment, tumor assessment was made by central radiology reviewers who were masked to assignment and data from patients. Tumor assessments performed during the open-label treatment period were only performed locally by the investigator/radiologist and were not reviewed centrally.

The clinical health states for this study were defined as follows.

- Baseline, progression-free (P0.0): QoL observations made on day 1 of cycle 1 before commencing blinded treatment.
- On-treatment, progression-free (P0.n): QoL observations made on day 1 of each cycle (every 4 weeks) during the first 3 months, and at the first day of every other cycle (every 8 weeks) thereafter.The QoL questionnaires were administered before the patient consulted a physician and before any study-related procedures were done.
- At first progression (P1.0): QoL observation made on day 1 of the cycle *at which first disease progression was confirmed* by central radiology review according to modified RECIST criteria (v 1.1);
- Post-first progression (P1.n): QoL observations made on day 1 of each cycle *following the cycle at which first progression was identified* (P1.0). During the treatment period (including open-label treatment), QoL observations were made at each cycle (every 4 weeks) during the first 3 months, then at the first day of every other cycle (every 8 weeks) thereafter.
- Second progression (P2): QoL observations made at or after any date following the date of second disease progression identified locally by the local investigator or their radiologist.

### Health utility assessment

This study focuses on one of the exploratory efficacy variables, the European Quality of Life group (EuroQol) five-item questionnaire (EQ-5D-3L) [20]. EQ-5D measures patient health utility (health status/QoL) using a descriptive system that assesses five generic dimensions of health: mobility, self-care, usual activity, pain and discomfort, and anxiety and/or depression. Each question has three possible responses that reflect the degree of impairment experienced by the patient on the day of assessment: no impairment (level 1), moderate problems (level 2), and extreme problems (level 3). The five health dimensions combine to form a descriptive health state (e.g., ‘11111’ = perfect health). In the current study these health states were converted to a single summary score, the EQ-5D index score, using the societal preference weighting algorithm for the United Kingdom (the default ‘tariff’ for EQ-5D) [21]. According to the EQ-5D index, 1.0 represents perfect health and 0.0 represents death.

The EQ-5D questionnaire was completed by patients at baseline (day 1 of cycle 1), at the first day of each cycle (every 4 weeks) during the first 3 months, at the first day of every other cycle (every 8 weeks) thereafter, and at the end of treatment visit. Questionnaires were completed at the start of each clinic visit, before the patient saw their physician and before any study-related procedure was conducted, so that any interaction between patients and physicians or other healthcare providers did not influence the responses to the questionnaires.

This primary outcome variable for this study is the EQ-5D index score.

### Sample size

One hundred and ninety-nine patients were randomized to receive double-blind treatment. This study presents results as of the primary completion date (26 January 2012), defined as when approximately 144 PFS events were observed. Patients were stratified at randomization according to (1) whether study treatment represented either third-line or fourth-line treatment (or beyond), and (2) geographic region (Asia vs. the rest of the world). Patients with both baseline EQ-5D assessment and at least one post-baseline assessment were included in the analysis.

### Statistical methods

Paired-samples *t* tests were used initially to assess within-subject difference in EQ-5D utility at baseline in the progression-free state (P0.0; day 1 of cycle 1) and the first post-progression observation (P1.1). The selection of first follow-up visit subsequent to the diagnosis of first disease progression, excluding observations made on the same day as progression was identified (and before patient knowledge of progression), was made to evaluate the impact of progression on the health utility including patient awareness of progression. Seventy-seven patients had paired observations at baseline and immediately following progression. Given a standard deviation for baseline utility of 0.221, a paired-samples *t* test would be able to detect a mean difference of 0.0715 utility with 95 % confidence and 80 % power [22]. This estimate is below the mean minimally important difference for the EQ-5D index (0.074 utility) [23], suggesting the current study would detect a clinically meaningful difference in utility should one exist.

Repeated-measures general linear mixed-effect modeling (GLMM) was also conducted, which correlated repeated observations from distinct patients using the EQ-5D index score as the dependent variable. A first-order, autoregressive covariance structure was chosen for repeated effects (allowing higher baseline values to correlate with related follow-up values); subject identity was modeled as a random effect. Fixed-effects factors and covariates included gender, age at baseline (in whole years), line of therapy (third-line or fourth-line or higher), treatment cycle number (as an integer), treatment modality at observation (regorafenib + BSC or placebo + BSC or off-treatment), and disease state [progression-free state (P0); state at first progression (P1.0); state following first progression (P1.n); and state following second progression (P2)]. Fixed-effect specifications were determined by manual inclusion of all other factors and covariates, with parameter significance *p* ≤ 0.05. Following determination of a main-effects structure, all two-way interactions were tested. The final model selected was that which maximized explained variance as indicated by Akaike’s information criterion (AIC; where smaller is better). Intermediate models included cycle number to test the assumption that utilities are stable across repeated treatment cycles while adjusting for disease progression status. The optimal final model was then used to calculate estimated marginal means for each of the target disease states.

The statistical evaluation was performed by using the software package SPSS release 20.0 [24].

## Results

### Recruitment

A total of 240 patients were enrolled; 41 patients (17.1 %) failed screening, and 199 patients (82.9 %) were randomized. Data cutoff was Jan 26, 2012 when the target 144 events were reached for the final PFS analysis. During the double-blind period, 38 patients (29 %) in the placebo group and 7 (11 %) patients in the regorafenib group discontinued study treatment. The most common reason for termination of study treatment was radiologically confirmed disease progression. As of data cutoff, 185 distinct patients had completed 803 EQ-5D questionnaires. Six hundred and twenty-four (77.7 %) observations were captured from patients in the P0 state (progression-free); 52 (6.5 %) were captured in state P1.0 (at progression), 112 (13.9 %) were captured in state P1.n (post-first progression), and 15 (1.9 %) were captured in state P2 (post-second progression). There were 79 patients in total with baseline and observations in the post-progression states (P1.n and P2).

### Losses and exclusions

There were no missing data from any of the EQ-5D questionnaires included in the analysis, and all observations were evaluable.

### Baseline data

Among the 182 subjects with EQ-5D observations at baseline and at least one post baseline, 117 (64.3 %) were male: average age was 58.0 years (SD 13.1) and mean body mass index (BMI) was 24.5 kg/m^2^ (SD 4.4). One hundred and two subjects (55.1 %) received study treatment as third-line, the remainder as fourth-line therapy or beyond. One hundred and twenty-three subjects (66.5 %) were randomized to receive regorafenib + BSC as their initial double-blind therapy, and 62 patients received placebo + BSC. Despite a slightly reduced patient count, all baseline characteristics closely reflected those of the intention-to-treat (ITT) population.

### Health state utility estimates

From the combined data set, the mean EQ-5D index score at baseline (day 1 of cycle 1) was 0.769 (SD 0.226). There were no significant between-group differences in baseline EQ-5D score (Table 1) for either treatment arm, line of therapy, or those among whom disease progression occurred.

**Table 1:** Comparisons of baseline EQ-5D index scores (day 1 of cycle 1) between study subgroups

Group	Baseline EQ-5D index			
	*n*	Mean	SD	*p*
Treatment arm				
Regorafenib 160 mg + best supportive care (BSC)	122	0.779	0.240	0.437
Placebo + BSC	60	0.751	0.195	
Line of therapy				
Third line	100	0.755	0.232	0.352
Fourth line and beyond	82	0.787	0.219	
Disease progression				
No	51	0.793	0.232	0.380
Yes	131	0.760	0.224	

### Paired-sample comparison

Seventy-seven patients had observations at baseline (P0.0) and at a clinic visit following confirmed first disease progression (P1.1). There was a statistically significant mean difference of −0.120 (*p* = 0.001; Table 2) between baseline- and first post-progression utility.

**Table 2:** Comparison of EQ-5D utility in first progression-free and post-progression states for patients whose disease progressed

	*n*	Mean	SD	SEM	*p*
P0.0 (first progression-free state)^a^	77	0.767	0.221	0.025	
P1.1 (first post-progression state)^b^	77	0.647	0.343	0.039	0.001

^a^Progression-free state represented by baseline observation

^b^Progression identified by independent final review (i.e., excludes any observation made on the same day as progression identified)

### Repeated-measures analysis

Preliminary modeling of single factors and covariates excluded gender, baseline age, and line of therapy as significant predictors of EQ-5D utility (data not shown). An intermediate main-effects model that included progression state, treatment cycle number, and treatment type reveals that while adjusting for progression state, neither cycle number (*p* = 0.341) nor treatment type (off-treatment vs. regorafenib, *p* = 0.749; placebo vs. regorafenib, *p* = 0.233) significantly influences observed utility (Table 3).

**Table 3:** Parameter estimates for general linear mixed model of EQ-5D utility with fixed effects of disease progression state, treatment cycle number, and treatment type with subject ID as a random effect

Parameter	Estimate	SE	d*f*	*t*	Significance	95 % CI of estimate	
						Lower	Upper
Intercept	0.741	0.020	305	36.884	0.000	0.701	0.780
Disease progression							
P0 (progression free)	Reference category						
P1.0 (at progression)	−0.032	0.028	708	−1.121	0.262	−0.087	0.024
P1.n (post-first progression)	−0.034	0.022	740	−1.526	0.127	−0.077	0.010
P2 (post-second progression)	−0.182	0.061	698	−2.962	0.003	−0.302	−0.061
Cycle number	−0.003	0.003	696	−0.952	0.341	−0.009	0.003
Treatment type							
Regorafinib + BSC	Reference category						
Placebo + BSC	0.037	0.031	335	1.194	0.233	−0.024	0.097
Off-treatment	−0.013	0.039	647	−0.321	0.749	−0.090	0.065

Removal of nonsignificant fixed effects leaves a model with one random effect [subject (random intercept and slope)] and one fixed effect, disease progression state. In this model the mean utility difference between the progression-free state (P0) and post-progression state (P1), at −0.041util, is smaller than that observed between the point estimates at baseline (P0.1) and first post-progression observation (P1.1), but of threshold statistical significance (*p* = 0.051). The mean utility for subjects following second disease progression (P2) was significantly lower than P0 at −0.231util (*p* < 0.001; Table 4). Corresponding estimated marginal mean utilities for each disease progression state are shown in Table 5.

**Table 4:** Parameter estimates for general linear mixed model of EQ-5D utility with fixed effects of disease progression state and with subject ID as a random effect

Parameter	Estimate	SE	d*f*	*t*	Significance	95 % CI of estimate	
						Lower	Upper
Intercept	0.743	0.016	206	46.781	0.000	0.712	0.775
Disease progression							
P0 (progression free)	Reference category						
P1.0 (at progression)	−0.034	0.028	708	−1.237	0.216	−0.089	0.020
P1.n (post-first progression)	−0.041	0.021	715	−1.958	0.051	−0.082	0.000
P2 (post-second progression)	−0.231	0.050	695	−4.651	0.000	−0.328	−0.133

**Table 5:** Estimated marginal means for EQ-5D utility by disease progression type (after model in Table 4)

Disease status	Mean	SE	d*f*	95 % CI of mean	
				Lower	Upper
P0 (progression free)	0.743	0.016	206	0.712	0.775
P1.0 (at progression)	0.709	0.029	755	0.652	0.767
P1.n (post-first progression)	0.703	0.023	575	0.657	0.748
P2 (post-second progression)	0.513	0.051	769	0.414	0.612

## Discussion

Advanced imatinib and sunitinib-resistant GIST is a condition often associated with relatively mild symptoms. In the analysis described here we discarded the EQ-5D assessed on the same date as progressive disease diagnosis. As per the clinical trial protocol, patients completed the EQ-5D before their clinical consultation that day at which progressive disease may have been identified. This finding would suggest that at the time of completing the EQ-5D, because of the lack of experience with worsened symptoms, many patients may not have been aware that they had progressed, which could influence their disease perception and consequently health state utilities. In fact, the difference between PF (P0.0) and utility at the time of progression (P1.0) was only −0.034 (*p* = 0.216). To capture both the physical and mental consequences of progressive disease, we report the first post-progression (P1.1) health state as it more likely reflects the impact of progressive disease.

Previous HRQoL investigations of patients with GIST were derived from the investigational randomized studies of sunitinib for patients with unresectable and/or metastatic GIST and who had failed or were resistant to imatinib. In the PF state, a utility value of 0.731 was observed for sunitinib-treated subjects and 0.781 for those on BSC [12]. The previously reported BSC utility values are consistent with the nonsignificantly different baseline utility values reported here for regorafenib and BSC of 0.779 and 0.751, respectively, as well as the utility value obtained from the combined PF data set of 0.769 (SD 0.226). The consistency of utility values between these two populations is surprising considering that the baseline population reported here had previously progressed on both imatinib and sunitinib, which could have suggested more severely impaired QoL.

The previous GIST utility assessment also noted differences in utilities between actively treated subjects and BSC [12]. An appraisal of these data noted that the specific disutility of treatment-related events was not reported; however, it was acknowledged that the impact of such events was implicit in the lower utility values for actively treated subjects. This result underscores the importance of understanding the impact of treatment-related adverse events on patients in an effort to balance benefits and safety.

The paired-samples comparison illustrates that, for those cases identified with disease progression by independent review, the incremental utility between progression-free (PF) and progression state to be 0.120 utilities, a statistically significant difference (*p* = 0.001). The observed difference between PF and progression was greater than the reported minimally important difference (MID) of 0.074 utilities for the UK tariff of the EQ-5D index, suggesting the difference is clinically meaningful in addition to being significantly different [23]. This analysis is strengthened by the finding that there was no difference in baseline utility between those who remained in a progression-free state and those who eventually progressed.

General linear models are an accepted approach for evaluating QoL when repeated observations for subjects are available over time [25]. As described here, the repeated-measures analysis provides the opportunity to assess variables that may impact health state utilities in the same subjects over time. As presented in Table 3, we indicate that utility remains stable in successive treatment cycles over time within a given health state. Furthermore, as describe here we report no significant difference in utility scores for PF regorafenib-treated patients compared to placebo (*p* = 0.233) within the same health state, suggesting similar QoL in the two groups. Furthermore, the lack of treatment effect across treatment groups supports the concept that the health states can be combined into a single data set for estimating utilities.

Based on the comparison of the primary and secondary analyses described here, the paired-samples estimate is likely the more robust estimate of utility for non-progressed and progressed health states for subjects with GIST. This finding is attributed to the protocol design that allowed placebo-treated subjects who are diagnosed with disease progression to pursue active treatment with regorafenib open label. Because there is substantial crossover of placebo subjects to active treatment, there is very limited opportunity to observe utility values for progressive disease in the presence of BSC, which reflects the current treatment scenario for patients with GIST. Consequently, during the crossover period the repeated measure would contain utility observations that occurred in the initial diagnosis of progressed disease, but then also the active treatment with regorafenib. Because of the crossover design the r epeated-measures analysis does not contain a homogeneous progressed population for estimating utility of these subjects. On this basis, the paired-sample analysis provides a better estimate of utility for both the progressed and non-progressed subjects.

The clinical trial investigation of regorafenib for GIST demonstrated significantly improved progression-free survival compared with BSC [18]. The results described here illustrate the preference for progressed and non-progressed GIST health states. The utility values reported here can be used to complement technology appraisals for reimbursement assessments in which utility values are used to construct quality-adjusted life-years (QALYs). Increasingly, QALYs are the preferred outcome measure of technology appraisal groups for making reimbursement assessments [15].
